# Traveling Before, During, and After the Pandemic: Impact of the COVID-19 Pandemic on the Travel Behavior and Travel Medicine Practice

**DOI:** 10.7759/cureus.66247

**Published:** 2024-08-05

**Authors:** Inês Figueiredo, Tiago Teixeira, Sofia Nunes, Cristóvão Figueiredo, Joana Fragoso, Carlos Azevedo, Diana Moreira, Luís Malheiro

**Affiliations:** 1 Medicine Department, Faculty of Medicine of University of Porto, Porto, PRT; 2 Travel Clinic, Unidade Local de Saúde de Gaia/Espinho, Vila Nova de Gaia, PRT; 3 Travel Clinic, Unidade Local de Saúde de Gaia/Espinho, Vila Nova De Gaia, PRT

**Keywords:** travel, tourism, travel medicine, immunization, covid-19

## Abstract

Objective: The emergence of the coronavirus disease 2019 (COVID-19) pandemic severely compromised international travel and the practice of travel medicine. This study aimed to investigate the evolution of traveler behaviors and prophylactic prescriptions across the pandemic and post-pandemic periods.

Population and methods: A retrospective study was conducted on travelers attending the International Vaccination Center in Vila Nova de Gaia, Portugal, from August 2019 to May 2023, where data were collected on travelers’ demographics, destination, duration, reasons for traveling, and data regarding travel-related vaccines and malaria prophylaxis. Travelers’ characteristics were compared between Period A (pre-pandemic), Period B (pandemic), and Period C (post-pandemic).

Results: The study included 1,711 travelers in the analysis. During the pandemic period, there were fewer travelers for tourism (5% decrease) and an increase in travelers for emigration and work (4.8% increase). There was also an increase in trips lasting less than two weeks among tourists, as well as trips lasting more than one month, primarily among travelers for work or emigration. During the pandemic, there was a significant decrease in Asia as a destination, with a shift toward African countries, which partially reverted in the post-pandemic period. Significant decreases in the prescriptions of vaccines were found during the pandemic and continued in the post-pandemic period.

Conclusion: There was a change in travelers' characteristics due to the pandemic, with a shift to shorter trips for tourism, an avoidance of Asia, and a preference for sub-Saharan African countries as a main hub of destination. Some vaccine prescription practices remained low and even decreased during or after the pandemic.

## Introduction

In late 2019, the emergence of coronavirus disease 2019 (COVID-19) in Asia marked the beginning of a global health crisis. Epidemiological tracing pinpointed Wuhan, China, as the initial epicenter, leading to an immediate international public health response as the virus rapidly spread [1].

Portugal felt the pandemic's impact early in 2020 with the rapid implementation of lockdowns and travel restrictions. As the COVID-19 pandemic compromised international travel, the practice of travel medicine and the tourism sector were widely affected [2,3].

At that time, several authors began questioning the future of travel medicine and how COVID-19 would change travelers’ behavior, especially for travelers at higher risk of severe disease, such as those with older age and underlying conditions [4,5]. During times of crisis, it has been described that travelers adapt their behavior according to their personality traits and in response to the perceived risks [3,6]. The fear of overcrowded places, the uncertainty of sudden lockdowns, and complexities in self-testing are pointed as reasons for shorter trips and more cautious tourist behavior [6]. These challenges emphasized the vital significance of travel medicine, which had to quickly adapt to the growing concerns over the risks associated with international travel during the pandemic.

Although it is expected that fear diminishes over time, it is unknown how the pandemic’s effects on travel behavior last among travelers. According to the United Nations World Tourism Organization (UNWTO) World Tourism Barometer, international tourism ended 2023 at 88% of pre-pandemic levels, with the highest recovery being recorded in Europe and Africa [7]. In a study performed during the pandemic in Malaysia, older participants were less prone to travel for leisure and visit friends and relatives early in the pandemic, and their risk perception and fear eased as the pandemic progressed and vaccines became more available to a wider population [6].

As the pandemic ended and international mobility increased, we question if there was a discernible change in travel behavior, destination choice, and an upsurge in health consultations, reflecting the public's need to understand and comply with new travel regulations. We also question whether vaccine fatigue due to COVID-19 may have altered the vaccine and antimalarial prescription practices in travel medicine. This study aimed to investigate the evolution of traveler behavior across the pre-pandemic, pandemic, and post-pandemic periods. We evaluated if there were declines in pre-travel consultations, changes in traveler characteristics such as age and comorbidities, and shifts in the reasons for traveling and destination preferences. We also explored if there were significant differences in prophylactic prescriptions such as antimalarial and travel-related vaccines.

## Materials and methods

Population and study design

We performed an observational retrospective study including travelers attending an International Vaccination Center (IVC) in Vila Nova de Gaia, Portugal, between August 2019 and May 2023. This study was approved by the institutional ethics committee (reference 52/2023), and a waiver of informed consent was obtained.

All travelers attending the IVC during the study period were considered for inclusion, and the only exclusion criterion was an incomplete or missing registry. The study population was divided into three: Period A (pre-pandemic), from the IVC opening on August 28, 2019, to March 17, 2020; Period B (pandemic) from March 18, 2020, to June 30, 2022; and Period C (post-pandemic) from July 1, 2022, to May 31, 2023. The IVC was closed due to the lockdown between March 18, 2020, and July 20, 2020. The first cut-off was defined as the moment when the state of emergency was declared in Portugal on March 18, 2020. The second cut-off was defined as the moment when the Portuguese government stopped requiring proof of test to screen for SARS-CoV-2 infection, presentation of COVID-19 EU digital certificate, vaccination, or recovery certificate to enter the country.

Data collection

Data were collected from the departments’ database used for internal audit. Anonymous traveler data are routinely introduced in the database after the appointment, which is used for annual audits on prescription practices. The database includes age group, sex, destination, duration, reason for traveling, special traveler characteristics (immunosuppression, pregnancy or breastfeeding, and prior allergic reaction), and prescription of a travel-related vaccine or malaria prophylaxis.

Age was categorized in 10-year intervals, except for infants less than 12 months of age who were considered separately and elderly travelers more than 70 years old who were grouped in one category. For study purposes, the destination was defined according to the WHO geographic regions and sub-regions and further characterized as a single destination or multi-country destination.

For study purposes, the research team considered a vaccine recommended when travelers were at risk. The specific criteria for malaria prophylaxis or a travel-related vaccine can be found in Table 1. Endemicity was defined according to the CDC Yellow Book: Health Information for International Travel or the World Health Organization and adapted to the time of travel [8,9]. The vaccine was considered not to be recommended when 1) the immunization criteria were not met, 2) the patient had been previously fully immunized and not a candidate for a booster dose, 3) there was a major contraindication to the vaccine, and 4) the vaccine was age-restricted.

**Table 1:** Criteria used to consider a vaccine as “recommended."

Type of intervention	Criteria used
Malaria prophylaxis	Travel to areas with ongoing malaria transmission taking into account individual risk assessment such as the itinerary, types of accommodations, season, style of travel and special health conditions (e.g., pregnancy).
Cholera vaccine	Travelers visiting areas of active cholera transmission.
Tick-borne encephalitis vaccine	Travelers moving to a tick-borne encephalitis endemic area or traveling for >2 weeks during spring or summer and planning on having outdoor activities where they may be in contact with ticks such as hiking, hunting or camping.
Japanese encephalitis vaccine	Long-term travelers (≥1 month) to a Japanese encephalitis area, according to seasonality. Short-term travelers (<1 month) to endemic areas with permanence in high-risk areas (such as camps, rural regions, rice paddies, etc.) according to seasonality.
Yellow fever vaccine	Travelers whose destination is a country at high risk of yellow fever transmission or whose destination is a country with a low risk of transmission but with high exposure to mosquito bites (such as extended stays to rural areas) or travelers passing through a country at high risk in need of a proof-of-vaccination.
Typhoid fever vaccine	Travelers to a typhoid fever endemic area with a continuous or intermittent stay longer than three weeks or a shorter stay in an endemic area with high-risk factors (i.e., backpacking, humanitarian mission, healthcare workers, outbreaks, visiting family and friends).
Hepatitis A vaccine	Travelers to areas of high hepatitis A endemicity, including Africa, Asia, the Mediterranean Basin, the Middle East, and Central and South America. Healthcare professionals on humanitarian missions or placed in endemic areas.
Hepatitis B vaccine	Long-term travelers staying in an endemic region, where exposure cannot be avoided in situations of accident or medical treatment. Visits to spouses who are staying for a long time in an endemic region.
Poliomyelitis vaccine (booster)	Travelers staying >4 weeks in countries with risk for poliovirus spread.
Tetravalent meningococcal conjugate vaccine	Travelers to parts of the meningitis belt of sub-Saharan Africa during the dry season (December–June). Pilgrims to the Umrah or Hajj.
Rabies pre-exposure prophylaxis	Travelers whose destination or travel characteristics fall under categories 1 to 4 according to the Yellow Book rabies preexposure prophylaxis recommendations.
Measles vaccine	Portuguese travelers born in or after 1971 without proof of previous vaccination or infection.

Statistical analysis

Traveler characteristics were examined across the three distinct periods. Descriptive statistics, including absolute frequencies and proportions within each period, as well as the total duration of the study, were calculated. Comparisons were conducted using the Pearson chi-square test at a 95% confidence interval, with adjustments made for the number of cells through the Bonferroni correction [10,11]. Data on COVID-19 vaccine rates in Portugal were taken from the national vaccination report [12].

To evaluate prescription practices across time, we considered the proportion of travelers for whom a specific vaccine was prescribed when recommended. Period A was considered the reference and compared to Periods B and C with a logistic regression. The results are presented as odds ratios (ORs) and p-values. The total number of prescriptions in each period was not considered for analysis as they were dependent on travel destination and patient characteristics, which could vary between periods. Data treatment and analysis were conducted using IBM SPSS statistics (IBM Corp. Released 2020. IBM SPSS Statistics for Windows, Version 27.0. Armonk, NY: IBM Corp).

## Results

General description

In total, 1,945 participants who attended the IVC at Centro Hospitalar de Vila Nova de Gaia/Espinho (CHVNGE) between August 28, 2019, and May 31, 2023, were identified through the institutional electronic medical records. We included 1,711 travelers who had complete data recorded in the internal audit database. Conversely, 234 travelers with incomplete or missing data were excluded from the study.

**Figure 1:**
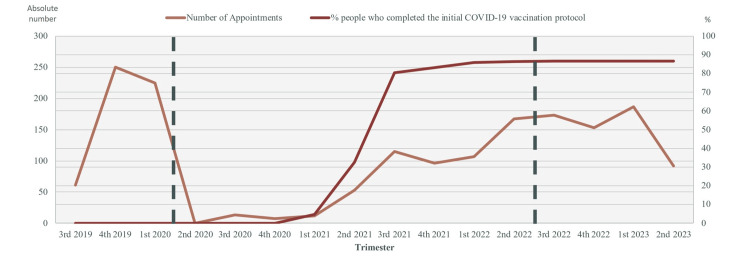
Temporal comparison of International Vaccination Center conducted appointments and completion of initial COVID-19 vaccination protocol by trimester. Data spans pre-pandemic, pandemic, and post-pandemic periods, demarcated by vertical dashed lines. Trimesters are indicated as 1st (January-March), 2nd (April-June), 3rd (July-September), and 4th (October-December). Data on COVID-19 vaccine rates in Portugal were taken from the national vaccination report [12].

Of the 1,711 included participants, 536 (31.3%) traveled in the pre-pandemic period, 570 (33.3%) traveled in the pandemic period, and 605 (35.4%) traveled in the post-pandemic period. The number of evaluated travelers per trimester across the study period is described in Figure 1. The mean number of evaluated travelers per month was 67 travelers/month in the pre-pandemic period, 21 travelers/month in the pandemic period, and 50 travelers/month in the post-pandemic period. The number of available appointments was similar during the three study periods, except between March 18, 2020, and July 20, 2020, when the travel clinic was closed due to pandemic-associated lockdowns.

Inter-period evaluation

Table 2 provides an overview of the characteristics of the travelers across study periods. No significant differences were observed in the sex or age distribution throughout the designated periods. During the pandemic period, fewer travelers had tourism as the reason for traveling, while there was an increase in the proportion of work/migration. In the post-pandemic period, traveling for tourism significantly increased and became the main motive for traveling, while the proportion of work/migration significantly decreased. No other differences were found in the proportion of travelers with other reasons for traveling. Throughout the three periods, trips with a duration under two weeks were the most common; although in the pre-pandemic period, trips with a duration between two and four weeks were also common (42%). During the pandemic, there were significantly fewer travelers with trips between two and four weeks (26.8% vs. 42%, period B vs. A), while those with trips longer than one month (19.7% vs. 12.9%, period B vs. A) significantly increased. In the post-pandemic period, this trend reverted, with fewer travelers with trips longer than one month (10.7%).

**Table 2:** Descriptive analysis of the travelers * Statistically significant differences were assessed using the Person chi-square test at a 95% confidence interval, accounting for the Bonferroni adjustment for the number of cells. NA: The Person chi-square test was deemed inapplicable due to a failure to meet the required assumptions. This occurred when the expected values did not reach 5 in more than 20% of the cells, or if a cell had fewer than one expected value. ^a^ Indicates significant differences from the expected values at the Bonferroni-adjusted alpha level, suggesting values lower than expected. ^b^ Indicates significant differences from the expected values at the Bonferroni-adjusted alpha level, suggesting values higher than expected.

	Period A (n = 536)	Period B (n = 570)	Period C (n = 605)	Total	p-value
	n (%)	n (%)	n (%)	n (%)	
Sex					0.084
Female	273 (50.9)	274 (48.1)	330 (54.5)	877 (51.3)	
Male	263 (49.1)	296 (51.9)	275 (45.5)	834 (48.7)	
Age					0.040
<1	7 (1.3)	7 (1.2)	9 (1.5)	23 (1.3)	
1_9	31 (5.8)	37 (6.5)	67 (11.1)	135 (7.9)	
10_19	22 (4.1)	32 (5.6)	18 (3.0)	72 (4.2)	
20_29	155 (28.9)	153 (26.8)	164 (27.1)	472 (27.6)	
30_39	148 (27.6)	146 (25.6)	157 (26.0)	451 (26.4)	
40_49	97 (18.1)	92 (16.1)	84 (13.9)	273 (16.0)	
50_59	49 (9.1)	74 (13.0)	69 (11.4)	192 (11.2)	
60_70	20 (3.7)	25 (4.4)	30 (5.0)	75 (4.4)	
≥71	7 (1.3)	4 (0.7)	7 (1.2)	18 (1.1)	
Reason for traveling					<0.001*
Tourism	334 (62.3)	324 (56.8)^a^	454 (75.0)^b^	1112 (65.0)	
Emigration and work	119 (22.2)	154 (27.0)^b^	70 (11.6)^a^	343 (20.0)	
Visiting family and friends	58 (10.8)	66 (11.6)	67 (11.1)	191 (11.2)	
Mission or volunteering	21 (3.9)	23 (4.1)	14 (2.3)	58 (3.4)	
Others	4 (0.7)	3 (0.6)	0	7 (0.4)	
Duration					<0.001*
Less than 2 weeks	242 (45.1)^a^	305 (53.5)	328 (54.2)	875 (51.1)	
2 to 4 weeks	225 (42.0)^b^	153 (26.8)^a^	212 (35.0)	590 (34.5)	
>1 to 6 months	51 (9.5)	71 (12.5)^b^	42 (6.9)	164 (9.6)	
More than 6 months	18 (3.4)	41 (7.2)^b^	23 (3.8)	82 (4.8)	
Special traveler characteristics					NA
Immunosuppression	9 (1.7)	9 (1.6)	14 (2.3)	32 (1.9)	
Prior allergic reaction	5 (0.9)	1 (0.2)	9 (1.5)	15 (0.9)	
Pregnancy or breastfeeding	4 (0.7)	1 (0.2)	5 (0.8)	10 (0.6)	
Continent					<0.001*
Africa	200 (37.3)^a^	375 (65.8)^b^	303 (50.1)	878 (51.3)	
Americas	118 (22.0)	128 (22.5)	125 (20.7)	371 (21.7)	
Asia	211 (39.4)^b^	53 (9.3)^a^	167 (27.6)	431 (25.2)	
Other continents	4 (0.8)	3 (0.6)	3 (0.5)	10 (0.6)	
Multiple continents	3 (0.6)	11 (1.9)	7 (1.2)	21 (1.2)	

To clarify the reasons behind this change in travel duration, a sub-analysis through a logistic regression was performed focusing on the motives for traveling and duration, comparing the pandemic and post-pandemic periods with the pre-pandemic period. The increase in trips longer than one month during the pandemic period was mainly due to a higher number of travelers for emigration and work (p = 0.001). In travelers for tourism, there was an increase in trips lasting less than two weeks (p = 0.001) and a reduction in trips with a duration of two to four weeks (p = 0.001). This trend partially persisted in the post-pandemic period, although there was a recovery in the proportion of trips between two and four weeks (p = 0.01). No differences in the proportion of travelers with severe vaccine allergy or who were immunosuppressed or pregnant were found as the Pearson chi-square test was inapplicable due to a failure to meet the required assumptions.

**Figure 2:**
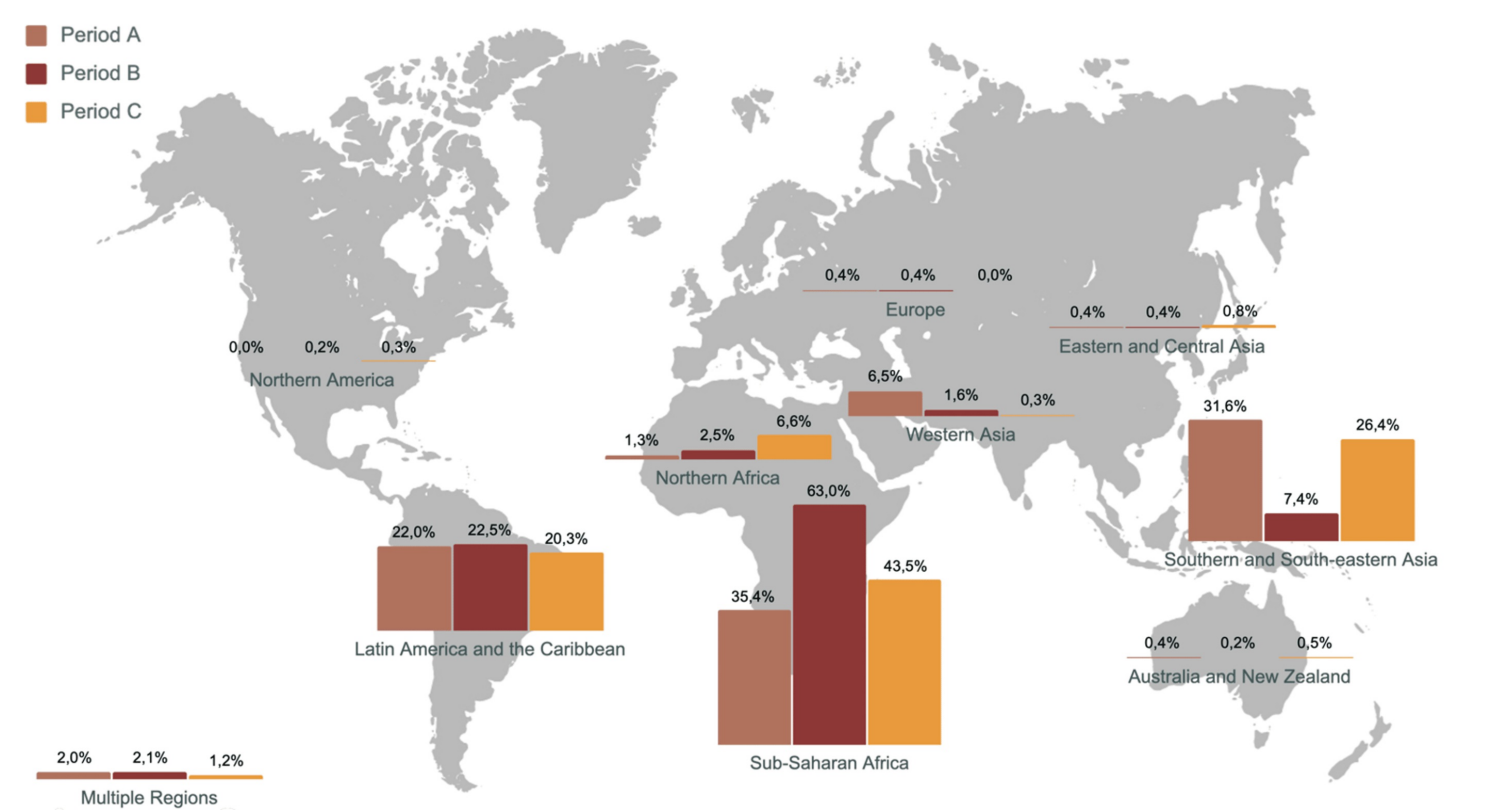
Traveler distribution by WHO sub-regions across the different periods of the COVID-19 pandemic. WHO's sub-regions were restructured into broader categories for the purposes of this analysis. "Europe" was treated as a single entity, combining the sub-regions of Eastern, Northern, Southern, and Western Europe. Similarly, "Eastern and Central Asia" was a fusion of Central and Eastern Asia, while "Southern and South-eastern Asia" brought together South-eastern and Southern Asia. Oceania was presented as one region, potentially including Australia, New Zealand, and the Pacific Islands. This approach was taken due to the relatively low number of travelers in these areas, providing a clearer visual representation of the data.

During the pandemic period, there was a significant decrease in the proportion of travelers whose destination was Asia and a significant increase in the proportion of travelers to African countries. Travelers to South and Central America were stable across periods, and travelers to Europe, Oceania, and North America were residual. Travelers’ distribution by the WHO sub-regions across the different periods can be found in Figure 2. A further sub-analysis found that this increase in African destinations during the pandemic period was equally found in travelers for emigration and work, travelers visiting family and relatives, and travelers for tourism.

Prescription habits

A total of 3,134 vaccines were prescribed to 1,500 (87.7%) travelers during the study period. Malaria prophylaxis was prescribed in 789 (46.1%) travelers. The number of travelers at risk that would benefit from a specific vaccine and malaria prophylaxis, and the trends in vaccine prescription across study periods can be found in Table 3.

**Table 3:** Prescription of vaccines, malaria prophylaxis, and number of travelers to whom a vaccine or prophylaxis was recommended. ACWY: tetravalent meningococcal conjugate; HA: hepatitis A; HB: hepatitis B; JE: Japanese encephalitis; OR: odds ratio; TBE: tick-borne encephalitis; TF: typhoid fever; YF: yellow fever; NA: not applicable due to a limited number of prescriptions and a low count of travelers at risk;^ a^: absolute number and proportion (%) of travelers who were prescribed a vaccines/prophylaxis in comparison to the number of travelers to whom a vaccine/prophylaxis should have been recommended; ^b^: absolute number and proportion (%) of travelers to whom a vaccine/prophylaxis should have been recommended in comparison to the total number of travelers during that period; *: statistically significant differences in a logistic regression.

	Period A (n = 536)		Period B (n = 570)				Period C (n = 605)			
	n (%) prescriptions^a^	n (%) recommended^b^	(n) % prescriptions^a^	n (%) recommended^b^	OR (B vs. A)	p	(n) % prescriptions^a^	n (%) recommended^b^	OR (C vs. A)	p
TBE vaccine	1 (100.0)	1 (0.2)	1 (50.0)	2 (0.4)	NA	NA	0	0	NA	NA
JE vaccine	16 (57.1)	28 (5.2)	4 (25.0)	16 (2.8)	0.250	0.045^*^	5 (33.3)	15 (2.5)	0.375	0.142
YF vaccine	119 (96.0)	124 (23.1)	174 (93.5)	186 (32.6)	0.609	0.364	191 (95.0)	201 (33.2)	0.803	0.694
TF vaccine	231 (62.9)	367 (68.5)	239 (59.2)	404 (70.9)	0.853	0.282	220 (55.0)	400 (66.1)	0.720	0.026^*^
HA vaccine	301 (56.9)	529 (98.7)	343 (60.9)	563 (98.8)	1.181	0.177	365 (61.0)	598 (98.8)	1.187	0.159
HB vaccine	10 (20.0)	50 (9.3)	19 (26.4)	72 (12.6)	1.143	0.416	21 (47.7)	44 (7.3)	3.652	0.005^*^
ACWY vaccine	75 (56.0)	134 (25.0)	55 (30.4)	181 (31.8)	0.343	<0.01^*^	47 (34.6)	136 (22.5)	0.415	<0.01^*^
Poliomyelitis vaccine booster	24 (42.9)	56 (10.4)	42 (52.5)	80 (14.0)	1.474	0.269	21 (32.3)	65 (10.7)	0.636	0.232
Rabies pre-exposure prophylaxis	0	310 (57.8)	0	267 (48.8)	NA	NA	5 (1.6)	313 (51.7)	NA	NA
Measles vaccine	55 (61.1)	90 (16.8)	32 (45.1)	71 (12.5)	0.522	0.044^*^	17 (29.3)	58 (9.6)	0.264	<0.01^*^
Malaria prophylaxis	178 (89.4)	199 (37.1)	294 (87.5)	336 (58.9)	0.826	0.500	221 (84.0)	263 (43.5)	0.621	0.095

**Figure 3:**
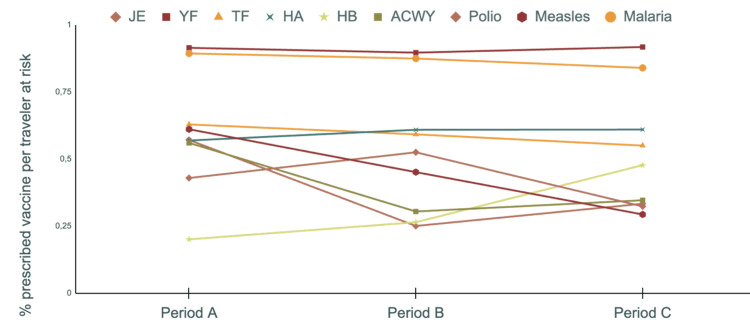
Proportion of travelers to whom a vaccine or malaria prophylaxis was prescribed per number of travelers to whom a vaccine/prophylaxis should have been recommended ACWY: meningococcal tetravalent conjugate vaccine, HA: hepatitis A vaccine, HB: hepatitis B vaccine, JE: Japanese Encephalitis vaccine, Polio: poliomyelitis vaccine booster, TF: typhoid fever vaccine, YF: yellow fever vaccine.

Prophylactic recommendations throughout time can be found in Figure 3. The most prescribed vaccines were hepatitis A (HA) (59.1%), typhoid fever (TF) (45.5%), and yellow fever (YF) (31.6%). The least prescribed vaccines were the cholera, rabies, and tick-borne encephalitis (TBE) vaccines, all prescribed to <1% of the travelers. Regarding the 511 travelers for whom the YF vaccine was recommended, the vaccine was mandatory in 144 cases (28.2%), and the proportions at which it was mandatory were not significantly different between periods A, B, and C (n = 33, 26.6%; n = 61, 32.8%; n = 50, 24.9%, respectively; p = 0.247 A vs. B, p = 0.727 A vs. C).

Adequate vaccine and antimalarial prophylaxis prescription according to indication ranged from 20% to 91.80%. Compared to the pre-pandemic period, in the pandemic period, there was a significant decrease in the proportion of prescribed vaccines for travelers to whom it was recommended. Significant decreases in the prescriptions of the Japanese encephalitis vaccine (JE) (75% decrease, p = 0.045), tetravalent meningococcal conjugate vaccine (ACWY) (65.7% decrease, p < 0.01), and measles vaccine (47.8% decrease, p = 0.044) were found. In the post-pandemic period, compared to the pre-pandemic period, there was a further decrease in measles vaccine prescription (73.6 % decrease, p < 0.01) and TF vaccine prescription (28.0% decrease, p = 0.026). The odds for hepatitis B (HB) vaccine prescription were 3.65 times higher in the post-pandemic period, being the only vaccine in which prescription significantly increased in comparison to the pre-pandemic period.

## Discussion

As expected, in our study, there was a significant decrease in travel consultations during and following the pandemic, reflecting the broad impact of the COVID-19 pandemic on travel. Concerning the purpose for traveling, we found a decrease in travel for tourism and, among those, a decrease in travel duration, while there was an increase in the proportion of travelers for emigration/work, which were associated with trips longer than a month.

We believe that these findings reflect the fear of COVID-19, the disruptions and expenses related to travel inconveniences and interruptions, a decrease in travel destination offers and commodities, difficulties with self-testing timings and vaccine certificates, and ongoing governmental restrictions. Fear of lack of money in a crisis and fear of infection during the trip have been pointed as two of the main factors predicting travel for all tourists [13]. In addition, frequent changes in travel regulations, ongoing concerns about new COVID-19 variants, and economic challenges might have led to travelers planning their trips with less lead time, possibly resulting in insufficient time to book travel medicine consultations. Mobility changes in response to COVID-19 have been well described, where significant reductions in travel duration under epidemic conditions mainly depended on the purpose of travel and means of transportation and the degree of fear of coronavirus [14]. As borders began to reopen and traveling was partially resumed, in 2021, there was a gradual return of traveler confidence and the number of consultations slowly started to increase. However, by the middle of 2022, the number of travelers attending the clinic was still staggered and below pre-pandemic levels. This decline reflects the overall reduction in international travel, with the United Nations World Tourism Organization (UNWTO) reporting a 72% and 71% drop in international travel in 2020 and 2021, compared with 2019 [7].

Furthermore, we noted a change in destination preferences during the pandemic period, with a significant decline in the percentage of travelers opting for Asia and an increase in those choosing African countries. This finding can be attributed to several factors as, early in the pandemic, Asia was perceived as the epicenter leading to heightened travel restrictions and concerns about the risk of infection. This perception, along with stringent lockdown measures and travel bans implemented by Asian countries, likely severely deterred travelers.

On the other hand, during the pandemic, African countries may have been perceived as having fewer COVID-19 outbreaks and fewer restrictions on incoming travelers compared to Asia. In addition, Africa has long been a primary destination for Portuguese emigrants, and notably, there was an increase in travel for work and emigration purposes during the pandemic [15]. It is also possible that the availability of travel options and flights influenced traveler behavior during this period, as African countries may have appealed to Portuguese travelers due to proximity, shorter flights, and easier returns in case of an emergency. Although the perceived risk of getting a respiratory infection during commercial flights seems to be greater than the actual risk, in terms of COVID-19 transmission during flights, flight duration is the most important parameter, while cabin disposition also seems to play a role, as most transmissions occur directly from an index case to the surrounding passengers before air filtration [16-18]. Nonetheless, this switch in destination is not devoid of risks for travelers, as the incidence of malaria due to *Plasmodium falciparum* and other vector-borne diseases such as yellow fever pose different risks from what would be expected in Asia.

In the post-pandemic period, we verified a partial recovery of Asia as a main travel destination, which reflects the loosening of COVID-19 restrictions. There was also a significant increase in tourism, which, once again, became the predominant purpose for traveling in those attending the clinic, reflecting a return to more typical traveling behaviors. However, among those traveling for tourism, trips shorter than two weeks were still predominant, suggesting that traveling behaviors are still affected three years after the pandemic.

Other relevant findings were the significant changes in many of the travel-associated vaccine prescriptions. An increase in the prescription of most travel-related vaccines was expected as the pandemic drew unprecedented public attention to vaccine-preventable diseases. Unfortunately, it also brought polarized attitudes toward vaccination, reflecting the need for effective health communication countering vaccine misinformation, and supporting vaccine-related shared decisions. Except for the hepatitis B vaccine, in which there was a significant increase in prescription, no other vaccine or prophylaxis prescription significantly improved in the pandemic or post-pandemic period. In fact, in the pandemic period, there was a surprising decrease in the prescription of typhoid fever and measles vaccines and malaria prophylaxis. This trend may be attributed to multiple factors. As attention was drawn to COVID-19, many other diseases were not as emphasized in social media and health communication, failing to increase awareness of other ongoing infectious threats in endemic countries. In fact, many outbreaks that included, but were not restricted to, dengue, typhoid fever, Japanese encephalitis, and measles were described during and following the pandemic, serving as a reminder that the other threats did not disappear [19-22]. In addition, "vaccine fatigue" became apparent among both travelers and doctors, a well-described inertia or inaction toward vaccine information or instruction due to perceived burden and burnout [23]. Moreover, travelers' hesitance to receive multiple vaccines, fueled by concerns about side effects, potential healthcare exposure, and unclear guidelines on vaccine co-administration, as well as misconceptions about the severity of diseases and the necessity for vaccination, further compounded the issue [23,24]. Vaccine shortages are a relevant problem in the European Union and probably aggravated during and after the pandemic [23,24]. Many travel-related vaccines have faced shortages after the pandemic such as the Japanese encephalitis, hepatitis A, measles, and typhoid fever vaccines [25,26]. Difficulties in supporting vaccine-associated costs amid the economic crisis after the pandemic may have also deterred travelers and doctors from acquiring/prescribing optional vaccines.

Even prior to the pandemic, there was a concern with the under-prescription of certain vaccines to travelers who were potentially exposed, not meeting optimal levels for preventive medicine. The examples of vaccines being prescribed in less or about 60% of the travelers with indication, such as hepatitis A and typhoid fever, may reflect that vaccine hesitancy and lack of awareness may have already been present before the pandemic and, for some vaccines, may have gotten worse. Nonetheless, yellow fever vaccine and antimalarial prophylaxis prescription did not change during or after the pandemic, probably reflecting the impact of what knowledge about these diseases has both in travelers and medical practitioners.

The biggest examples of lack of disease awareness are probably the tick-borne encephalitis virus and rabies. Curiously, only 0.23% of assessed travelers had Europe as their destination, a figure that starkly contrasts with the popularity of European destinations among Portuguese travelers and results in a low number of patients considered at risk for TBE [27]. This discrepancy may be due to a sense of safety fostered by harmonized travel measures, which possibly diminish the perceived necessity for pre-travel consultations. Regarding rabies vaccine, the risk of being exposed to rabies in international travel has been shown to be more frequent than what is believed in common knowledge, with incidence rates of potential travel-related rabies exposures of four per 1000 travelers or attack rates of 2% per trip [28]. This risk is currently considered higher than the risk of acquiring yellow fever or typhoid fever during international travel [29]. International surveys have revealed that the perceived risk of exposure to rabies or TBE among travelers to endemic regions underestimates the need to receive pre-travel vaccinations [30]. Together, it underscores the need for increased awareness and proactive health measures, particularly for diseases that travelers may not commonly consider.

The strengths of our study include the systematic nature of collected data, which reflected the profile of most travelers attending the clinic, with very few excluded travelers due to lack of information. We included a significant number of travelers, during a prolonged period, which increases the power of our study. Furthermore, we evaluated three relevant periods, which allowed comparisons with statistical power. However, being a single-center study, our population probably reflects travelers living in the geographic area surrounding the hospital and may differ in beliefs, socioeconomic conditions, and medical literacy when compared to other centers. As we only considered the Portuguese time frames for study purposes, broader international timelines may have also influenced the number and destination of travelers. In addition, our data only reflects the search for a medical consultation and not the real number of travelers at risk, as many may have traveled without a travel consultation or consulted with their general practitioner. This may have induced a bias, as travelers with comorbidities may have avoided the travel clinic due to fear of getting sick, as it is integrated into a hospital center. Moreover, we only had access to whether the vaccine or antimalarial prophylaxis was prescribed or not, but we cannot confirm that the traveler received the vaccine or took the antimalarial prophylaxis, nor can we confirm the reasons behind the decision to vaccinate or not. Furthermore, although our travel clinic has six dedicated health professionals, we cannot exclude a possible bias in prescription as, although most follow the same guidelines, individual opinions, and experiences may account for differences in prescription practices. It was not possible to ascertain the reasons why certain vaccines were not prescribed when they were recommended. The evaluation of a traveler's disease risk was based only on their destination, type of travel, age, and travel duration, as we did not have information on planned activities or personal risk factors. This might have led to overestimating the number of people at risk, especially for the rabies vaccine, since we could not account for specific travel details or activities that could change exposure risks.

## Conclusions

After the first months of the pandemic, traveling resumed and slowly increased to pre-pandemic numbers. However, the pandemic changed travelers’ behavior, with a shift to shorter trips for tourism, an avoidance of Asia, and an increase in African countries as a main hub of destinations. This shift may pose new risks to travelers as many of the chosen countries are endemic for *Plasmodium falciparum* malaria and yellow fever. Even though the pandemic brought awareness to vaccination, some vaccine prescription practices remained low and even decreased during and after the pandemic.

Our results should help prepare mechanisms for fighting vaccine hesitancy in travelers and practitioners and help practitioners tailor health advice to create awareness of the infectious risks during and after a pandemic.
